# Pre-treatment vitamin D insufficiency predicts severe paclitaxel-induced sensory neuropathy in breast cancer patients: a prospective cohort study

**DOI:** 10.1038/s41598-026-50367-8

**Published:** 2026-05-05

**Authors:** Amany M. Elfeky, Muhammad I. El-Masry, Amr A. Mahmoud, Sara A. Amin, Rana El Falah, Mo’men M. Saadoun

**Affiliations:** 1 Clinical oncology and nuclear medicine, Faculty of Medicine, Kafrelsheikh University, Kafrelsheikh, Egypt; 2 Clinical Pathology, Faculty of Medicine, Kafrelsheikh University, Kafrelsheikh, Egypt; 3 Clinical oncology and nuclear medicine, Faculty of Medicine, Tanta University, Tanta, Egypt

**Keywords:** Vitamin D insufficiency, Paclitaxel, Chemotherapy-induced peripheral neuropathy, Breast cancer, Neuroprotection, Risk factors, Cancer, Diseases, Medical research, Oncology

## Abstract

Paclitaxel-induced peripheral neuropathy (CIPN) is a debilitating side effect affecting up to 70% of patients receiving paclitaxel chemotherapy, with severe symptoms occurring in approximately 30%. While non-modifiable risk factors such as age and genetics have been established, the role of modifiable factors, including vitamin D insufficiency, remains poorly characterized. This study aimed to evaluate the association between pre-treatment vitamin D levels and the incidence of severe CIPN in breast cancer patients receiving paclitaxel-based chemotherapy. A prospective cohort study was conducted on 300 breast cancer patients (stage I-III) receiving paclitaxel-based chemotherapy (80 mg/m²) for 12 weeks at Kafrelsheikh University Hospitals. Baseline serum 25(OH)D was quantified using the Elecsys Vitamin D Total II assay on the Cobas e411 platform, standardized to the VDSP reference method. Pre-treatment vitamin D levels were measured using liquid chromatography-tandem mass spectrometry (LC-MS/MS), with insufficiency defined as ≤ 20 ng/mL. CIPN was assessed using the European Organisation for Research and Treatment of Cancer Quality of Life Questionnaire-Chemotherapy-Induced Peripheral Neuropathy 20-item scale (EORTC QLQ-CIPN20), with particular focus on grade 3–4 sensory neuropathy. Statistical analyses included receiver operating characteristic (ROC) curves and multivariate logistic regression to identify independent risk factors. The mean pre-treatment vitamin D level was 23.45 ± 8.3 ng/mL, with 39.3% (118/300) of patients classified as having vitamin D insufficiency. Patients with vitamin D insufficiency demonstrated significantly higher rates of grade 3–4 sensory CIPN compared to those with sufficient levels (32.2% vs. 5.5%, *p* < 0.001). Mean vitamin D levels were significantly lower in patients who developed severe CIPN (17.5 ± 4.9 ng/mL vs. 24.6 ± 8.4 ng/mL, *p* < 0.001). ROC analysis demonstrated vitamin D’s predictive value for motor CIPN (AUC = 0.747, *p* = 0.038). Multivariate logistic regression analysis confirmed vitamin D insufficiency as an independent predictor of sensory CIPN (OR = 6.72, 95% CI: 3.09–14.61, *p* < 0.001), even after adjusting for age, body mass index, and treatment schedule. Vitamin D insufficiency is independently associated with an increased risk of severe paclitaxel-induced peripheral neuropathy in breast cancer patients. While causal inference cannot be drawn from this observational design, these findings provide a strong rationale for future randomized controlled trials to evaluate whether vitamin D supplementation could serve as a candidate preventive strategy to mitigate CIPN severity and optimize cancer therapy outcomes.

**Supplementary Information:**

The online version contains supplementary material available at 10.1038/s41598-026-50367-8.

## Introduction

Paclitaxel is a widely utilized and highly effective chemotherapeutic agent in the treatment of breast cancer, including hormone receptor-positive, HER2-positive, and triple-negative subtypes, with demonstrated efficacy in both adjuvant and metastatic settings^1^. Paclitaxel exerts its antineoplastic effect by binding to β-tubulin subunits of microtubules, promoting their polymerization and stabilization, thereby inhibiting mitotic spindle dynamics and arresting cell division at the G2/M phase. In non-metastatic breast cancer, a regimen of 80 mg/m² administered weekly for 12 weeks has become standard practice as part of multimodal therapeutic protocols^2^. Despite the favorable risk-benefit profile of weekly dosing compared to three-weekly schedules, many patients experience dose-limiting toxicities, with peripheral neuropathy representing the most clinically significant adverse effect^3^. Notably, the same mechanism of microtubule stabilization that underlies paclitaxel’s antitumor activity is thought to contribute to its neurotoxicity, as disruption of axonal transport in peripheral neurons leads to distal axonal degeneration.

Chemotherapy-induced peripheral neuropathy (CIPN) is a major treatment-limiting toxicity associated with multiple anti-cancer agents, particularly taxanes such as paclitaxel. Current literature indicates that approximately 70% of patients treated with paclitaxel develop some degree of peripheral neuropathy, with nearly 30% experiencing severe symptoms classified as grade 3 or higher^4^. The clinical impact of CIPN extends beyond the treatment period, as symptoms can persist for months to years following chemotherapy completion^5,6^, substantially compromising patients’ quality of life and functional capacity^7^.

Previous research has identified several non-modifiable risk factors for CIPN, including advanced age, ethnicity, and genetic polymorphisms in drug metabolism pathways^8^. More recently, attention has shifted toward potentially modifiable risk factors such as diabetes mellitus, sedentary lifestyle, obesity, and high cumulative paclitaxel exposure^8^. However, the role of nutritional deficiencies as modifiable predictive biomarkers for paclitaxel-induced neuropathy remains relatively unexplored.

The assessment methods for identifying and grading CIPN severity in both clinical trials and medical practice can be broadly categorized into patient-reported outcome instruments, composite scoring systems with functional assessment components, and quality-of-life tools^9^. The most commonly utilized assessment tool is the clinician-led National Cancer Institute-Common Terminology Criteria for Adverse Events (NCI-CTCAE), although other instruments, such as the Eastern Cooperative Oncology Group (ECOG) criteria and the World Health Organization (WHO) neurotoxicity scale, are also employed^10^. The current version of NCI-CTCAE (version 5.0) grades both motor and sensory neuropathy according to severity: asymptomatic (grade 1), moderate (grade 2), severe (grade 3), or life-threatening (grade 4) neurotoxicity^9^.

Baseline nutrient deficiencies may represent modifiable predictive biomarkers for paclitaxel-induced peripheral neuropathy, though these relationships remain inadequately characterized. Deficiencies of folic acid, B vitamins, and vitamin D are established risk factors for peripheral neuropathy in other disease states, including chronic alcoholism and diabetes mellitus^11^. Despite these known associations across different etiologies, the role of micronutrient deficiencies as risk factors for chemotherapy-induced neuropathy has not been well defined. Several retrospective studies have suggested that patients with lower pre-treatment vitamin D concentrations may have an elevated risk of CIPN^12^; however, prospective validation studies are lacking.

The discovery of effective preventative strategies to reduce paclitaxel-induced peripheral neuropathy represents a critical unmet clinical need. Validation of vitamin D insufficiency as a CIPN risk factor is an essential step toward developing evidence-based interventional strategies to prevent CIPN development, potentially improving treatment completion rates and clinical outcomes in breast cancer patients undergoing taxane-based chemotherapy.

### Study objectives

The primary objective of this study was to assess the relationship between pre-treatment vitamin D insufficiency and the development of grade 3–4 chemotherapy-induced peripheral neuropathy in breast cancer patients receiving paclitaxel-based chemotherapy. Secondary objectives included evaluating vitamin D’s predictive value for CIPN severity and identifying additional risk factors contributing to neuropathy development.

## Materials and methods

### Study design and setting

This prospective cohort study was conducted at the Oncology Department of Kafrelsheikh University Hospitals, Egypt, between July 2022 and July 2024. The study protocol was approved by the Kafrelsheikh University Institutional Review Board (KFSIRB200-290), and all procedures were performed in accordance with the Declaration of Helsinki and Good Clinical Practice guidelines. Each participant was assigned a unique identification code, and all personal data were maintained with strict confidentiality. Written informed consent was obtained from all patients after a comprehensive explanation of the study objectives, procedures, potential benefits, and risks. Study results are to be used solely for scientific and educational purposes.

### Patient population

This study enrolled 300 patients with histopathologically confirmed breast cancer who were scheduled to receive paclitaxel-based chemotherapy. All patients completed a minimum follow-up period of 12 weeks during active paclitaxel treatment, with extended monitoring for up to 24 months post-treatment. Clinical, pathological, and treatment-related data were prospectively collected and analyzed.

Eligibility criteria

Inclusion criteria:

- Histopathologically confirmed breast cancer (stage I-III).
- Age ≥ 18 years.
- Planned treatment with paclitaxel-based chemotherapy 80 mg/m² for 12 weeks.
- Eastern Cooperative Oncology Group (ECOG) performance status ≤ 2^13^.
- Adequate hematologic function: absolute neutrophil count > 2 × 10⁹/L, platelet count ≥ 100 × 10⁹/L, hemoglobin ≥ 10 g/dL.
- Adequate hepatic function: total bilirubin ≤ upper limit of normal (ULN), aspartate aminotransferase (AST) and alanine aminotransferase (ALT) ≤ 2.5× ULN.
- Adequate renal function: serum creatinine < 1.5× ULN.
- Adequate cardiac function without pre-existing conditions that would contraindicate chemotherapy administration.
- Ability to understand and provide written informed consent.

Exclusion criteria:

- Recurrent or metastatic (stage IV) breast cancer.
- Pregnancy or lactation.
- Pre-existing peripheral neuropathy from any cause (grade ≥ 1).
- Dementia, altered mental status, or psychiatric conditions that would prohibit understanding or provision of informed consent.
- Concurrent secondary malignancy.
- Medical conditions known to cause peripheral neuropathy (uncontrolled diabetes mellitus, chronic alcoholism, HIV infection, hereditary neuropathies, vitamin B12 deficiency, thyroid disorders).
- Current use of vitamin D supplementation exceeding 1000 IU daily.
- Previous treatment with neurotoxic chemotherapy agents.

### Treatment protocol

#### Surgical management

All patients underwent primary surgical treatment consisting of either breast-conserving surgery (lumpectomy with axillary lymph node assessment) or modified radical mastectomy, based on tumor characteristics and patient preference. Tumor tissue specimens (minimum 1 cm² of viable tumor) were submitted for comprehensive histopathological and immunohistochemical analysis, including estrogen receptor (ER), progesterone receptor (PR), and human epidermal growth factor receptor 2 (HER2) status determination.

#### Chemotherapy regimen

The chemotherapy protocol consisted of two sequential phases:

1. Anthracycline phase: Doxorubicin 60 mg/m² plus cyclophosphamide 600 mg/m² administered intravenously every 21 days for four cycles (AC×4).
2. Taxane phase: Paclitaxel 80 mg/m² administered intravenously weekly for 12 weeks (12 weekly doses) OR docetaxel 100 mg/m² every 3 weeks for four cycles.

Treatment modifications (dose reductions or delays) were implemented according to institutional guidelines based on toxicity severity.

#### Radiation therapy

Adjuvant radiation therapy was administered using three-dimensional conformal radiation therapy (3D-CRT) technique. Patients who underwent breast-conserving surgery received whole breast irradiation at a total dose of 50 Gy delivered in 25 fractions (2 Gy per fraction, 5 days per week over 5 weeks), followed by a tumor bed boost of 10 Gy in 5 fractions. Patients who underwent modified radical mastectomy with node-positive disease received post-mastectomy radiation therapy to the chest wall and regional lymph nodes according to National Comprehensive Cancer Network (NCCN) guidelines^14^.

### Vitamin D assessment

#### Sample collection and timing

Blood samples for vitamin D measurement were collected at baseline, prior to initiation of chemotherapy. Samples were obtained via venipuncture, processed within 2 h of collection, and serum was separated and stored at -80 °C until batch analysis.

#### Laboratory analysis

Baseline venous blood samples were collected prior to the first chemotherapy cycle. Serum 25(OH)D concentrations were quantified using the Elecsys Vitamin D Total electrochemiluminescence immunoassay (ECLIA) on the fully automated Cobas e411 analyzer (Roche Diagnostics, Mannheim, Germany). This assay employs a competitive binding principle and is standardized against the isotope dilution liquid chromatography-tandem mass spectrometry (ID-LC-MS/MS) Reference Measurement Procedure, adhering to the Vitamin D Standardization Program (VDSP) protocols. The assay demonstrated an intra-assay and inter-assay coefficient of variation (CV) of < 5% and < 7%, respectively, with a lower limit of detection of 3 ng/mL.

#### Clinical definitions

Participants were stratified into three mutually exclusive groups based on serum 25(OH)D levels.

- Deficiency: ≤12 ng/mL.
- Insufficiency: 12.1–20 ng/mL.
- Sufficiency: >20 ng/mL.

The primary outcome was vitamin D insufficiency (defined as serum levels ≤ 20 ng/mL).

### Peripheral neuropathy assessment

#### Assessment tool

Peripheral neuropathy was evaluated using the European Organisation for Research and Treatment of Cancer Quality of Life Questionnaire-Chemotherapy-Induced Peripheral Neuropathy 20-item scale (EORTC QLQ-CIPN20).^15^ This validated patient-reported outcome instrument quantifies sensory (9 items), motor (8 items), and autonomic (3 items) neuropathy symptoms experienced during the previous week, with each item scored on a 4-point Likert scale ranging from 1 (“not at all”) to 4 (“very much”).

#### Assessment schedule

The EORTC QLQ-CIPN20 was administered at the following time points.

- Baseline (prior to chemotherapy initiation).
- Weekly during paclitaxel treatment (weeks 1–12).
- At treatment completion.
- Follow-up assessments at 1, 3, 6, 12, and 24 months post-treatment.

#### Primary analysis focus

Given that paclitaxel predominantly causes sensory peripheral neuropathy, the 9-item sensory subscale was utilized as the primary outcome measure in the main analysis.^16–18^ The sensory subscale raw scores were linearly transformed to a 0–100 scale according to the standard EORTC scoring manual. To translate these patient-reported outcome scores into clinical severity grades, mapping strictly followed the validated concordance thresholds published by Lavoie Smith et al. (2013). Based on this validation, a transformed sensory score of < 30 corresponds to NCI-CTCAE Grade 1; scores from 30 to 69 correspond to Grade 2; scores from 70 to 82 correspond to Grade 3 (severe symptoms limiting self-care activities of daily living); and scores ≥ 83 correspond to Grade 4.

Neuropathy severity classification:

- Grade 1: Asymptomatic, clinical, or diagnostic observations only.
- Grade 2: Moderate symptoms, limiting instrumental activities of daily living.
- Grade 3: Severe symptoms, limiting self-care activities of daily living.
- Grade 4: Life-threatening consequences, urgent intervention indicated.

### Study endpoints

Primary endpoint: The primary endpoint was the incidence of grade 3–4 chemotherapy-induced sensory peripheral neuropathy at any time during the 12-week paclitaxel treatment period.

Secondary endpoints:

1. Incidence of grade 3–4 motor peripheral neuropathy.
2. Time to onset of grade 3–4 peripheral neuropathy.
3. Association between vitamin D levels and treatment modifications (dose reductions or delays) due to peripheral neuropathy.
4. Persistence of peripheral neuropathy symptoms at 6, 12, and 24 months post-treatment.

### Statistical analysis

All data were compiled in a centralized database and analyzed using SPSS Statistics version 26.0 (IBM Corp., Armonk, NY, USA) and R version 4.2.0 (R Foundation for Statistical Computing, Vienna, Austria). A professional biostatistician affiliated with Tanta University Hospital performed the statistical analyses.

#### Descriptive statistics

Continuous variables are presented as the mean ± standard deviation (SD) for normally distributed data or as the median with interquartile range (IQR) for non-normally distributed data. Categorical variables are presented as frequencies and percentages.

#### Comparative analyses

- Independent samples t-tests or Mann-Whitney U tests were used to compare continuous variables between groups, as appropriate.
- Chi-square tests or Fisher’s exact tests were employed for categorical variables.
- A two-tailed *p*-value < 0.05 was considered statistically significant.

#### Receiver operating characteristic (ROC) curve analysis

ROC curves were constructed to evaluate the predictive performance of vitamin D levels for grade 3–4 peripheral neuropathy. Area under the curve (AUC), optimal cutoff values, sensitivity, and specificity were calculated with 95% confidence intervals.

#### Univariate and multivariate analyses

Logistic regression models were constructed to identify risk factors for grade 3–4 sensory peripheral neuropathy. Variables with *p* < 0.10 in univariate analysis were included in the multivariate model. Results are presented as odds ratios (OR) with 95% confidence intervals (CI).

#### Sample size calculation

Based on previous retrospective data suggesting a CIPN rate of approximately 30% in patients with vitamin D sufficiency and 60% in those with insufficiency, a sample size of 270 patients (135 per group) was calculated to provide 80% power to detect this difference with a two-sided alpha of 0.05. Accounting for a potential 10% dropout, 300 patients were enrolled.

## Results

### Patient characteristics and treatment delivery

A total of 300 breast cancer patients meeting the eligibility criteria were enrolled and completed the study. The cohort was relatively young, with a mean age of 40.6 ± 9.8 years, and predominantly overweight or obese (mean BMI 29.4 ± 5.4 kg/m²) (Table 1). Regarding treatment schedules, the population was nearly evenly stratified: 153 patients (51.0%) received weekly paclitaxel, while 147 (49.0%) received the regimen every two weeks (Table 2). The delivered dose intensity was high, with a mean of 90.4% of the planned dose administered. However, neurotoxicity necessitated treatment modifications (dose reduction or delay) in 19.0% of the cohort.

**Table 1:** Baseline demographic and clinical characteristics (*N* = 300).

Characteristic	Value
Age (years), mean ± SD	40.6 ± 9.8
Body mass index (kg/m²), mean ± SD	29.4 ± 5.4
Menopausal status, n (%)	
Premenopausal	137 (45.7)
Postmenopausal	163 (54.3)
Tumor stage, n (%)	
Stage I	52 (17.3)
Stage II	156 (52.0)
Stage III	92 (30.7)
Nodal status, n (%)	
Node-negative	81 (27.0)
1–3 positive nodes	111 (37.0)
≥4 positive nodes	108 (36.0)
Hormone receptor positive (ER and/or PR), n (%)	210 (70.0)
HER2-positive, n (%)	63 (21.0)

**Table 2:** Treatment characteristics and vitamin D status (*N* = 300).

Variable	Value
Paclitaxel schedule, n (%)	
Weekly (80 mg/m²)	153 (51.0)
Every 2 weeks	147 (49.0)
Delivered paclitaxel dose (% of planned), mean ± SD	90.4 ± 5.0
Treatment modification due to CIPN, n (%)	57 (19.0)
Baseline vitamin D (ng/mL), mean ± SD	23.45 ± 8.3
Vitamin D status, n (%)	
Deficiency (≤ 12 ng/mL)	30 (10.0)
Insufficiency (≤ 20 ng/mL)	118 (39.3)
Sufficiency (> 20 ng/mL)	182 (60.7)

### Vitamin D status and neuropathy incidence

Baseline vitamin D assessment revealed a high prevalence of suboptimal levels; 39.3% of patients were classified as insufficient (≤ 20 ng/mL) and 10.0% as deficient (≤ 12 ng/mL) (Table 2), with a mean serum concentration of 23.45 ± 8.3 ng/mL. The incidence of grade 3–4 sensory CIPN was 16.0% (48/300), representing the primary dose-limiting toxicity (Table 3). Severe motor neuropathy was rare, occurring in only 2.0% of patients. Specifically, the incidence of severe sensory CIPN was 32.2% (38/118) in the vitamin D insufficient group versus 5.5% (10/182) in the sufficient group.

**Table 3:** Incidence of Chemotherapy-Induced Peripheral Neuropathy.

Neuropathy Outcome	*n* (%)
Grade 3–4 sensory CIPN	48 (16.0)
Grade 3–4 motor CIPN	6 (2.0)
Any grade 3–4 CIPN	51 (17.0)
Grade 1–2 sensory CIPN	189 (63.0)
No clinically significant CIPN	60 (20.0)

### Determinants of severe sensory neuropathy

Univariate analysis identified significant associations between severe sensory CIPN and clinical variables. Patients who developed grade 3–4 sensory neuropathy had significantly lower mean vitamin D levels compared to those who did not (17.5 ± 4.9 ng/mL vs. 24.6 ± 8.4 ng/mL; *p* < 0.001). In the updated multivariate logistic regression model adjusted for age, BMI, menopausal status, cumulative delivered dose, and delivered dose intensity (Table 4), vitamin D insufficiency emerged as the strongest independent predictor of severe sensory CIPN (Adjusted OR = 6.72, 95% CI: 3.09–14.61, *p* < 0.001). The treatment schedule was also a significant independent risk factor; patients receiving paclitaxel every two weeks had a 2.5-fold increased risk of severe neuropathy compared to those on the weekly schedule (Adjusted OR = 2.50, *p* = 0.009). Postmenopausal status showed a borderline association (Adjusted OR = 1.88, *p* = 0.075).

**Table 4:** Factors associated with Grade 3–4 Sensory CIPN and multivariate logistic regression analysis.

Variable	Grade 3–4 CIPN (*n* = 48)	No Severe CIPN (*n* = 252)	Adjusted OR (95% CI)	*p*-value
Vitamin D insufficiency (≤ 20 ng/mL), n (%)	38 (79.2)	80 (31.7)	**6.72 (3.09–14.61)**	**< 0.001**
Paclitaxel every 2 weeks, n (%)	34 (70.8)	113 (44.8)	**2.50 (1.25–5.08)**	**0.009**
Postmenopausal status, n (%)	34 (70.8)	129 (51.0)	1.88 (0.94–3.78)	0.075
Cumulative delivered dose (mg/m²)	Mean 847.3	Mean 791.6	1.004 (1.001–1.008) per mg/m²	**0.028**
Delivered dose intensity (% of planned)	88.1 ± 5.8	90.8 ± 4.8	0.94 (0.88–1.01) per 1%	0.11
Age (per year increase)	—	—	1.03 (0.99–1.06)	0.15
BMI (per kg/m² increase)	—	—	1.01 (0.95–1.07)	0.74

Percentages in this table represent column percentages (prevalence of the factor within each outcome group). For clinical incidence rates (risk of developing severe CIPN based on vitamin D status), please refer to the Abstract and Sect.  3.2, which report incidence rates of 32.2% in the insufficient group versus 5.5% in the sufficient group.

Bold values indicates statistical significance (*p* < 0.05).

### Sensitivity analysis for treatment heterogeneity

To ensure that the observed associations were not confounded by treatment schedule heterogeneity, a sensitivity analysis was conducted restricting the cohort exclusively to patients receiving the standard weekly paclitaxel regimen (*n* = 153). Within this homogenous subgroup, the multivariate model confirmed that vitamin D insufficiency remained a potent independent predictor of grade 3–4 sensory CIPN (Adjusted OR = 5.84, 95% CI: 2.11–11.45, *p* = 0.004) (Supplementary Table S1).

### Predictive value of vitamin D (ROC analysis)

Receiver operating characteristic (ROC) curve analysis was conducted to evaluate the ability of baseline serum 25-hydroxyvitamin D [25(OH)D] levels to discriminate patients who developed severe (grade 3–4) chemotherapy-induced peripheral neuropathy during paclitaxel treatment.

**Fig. 1:**
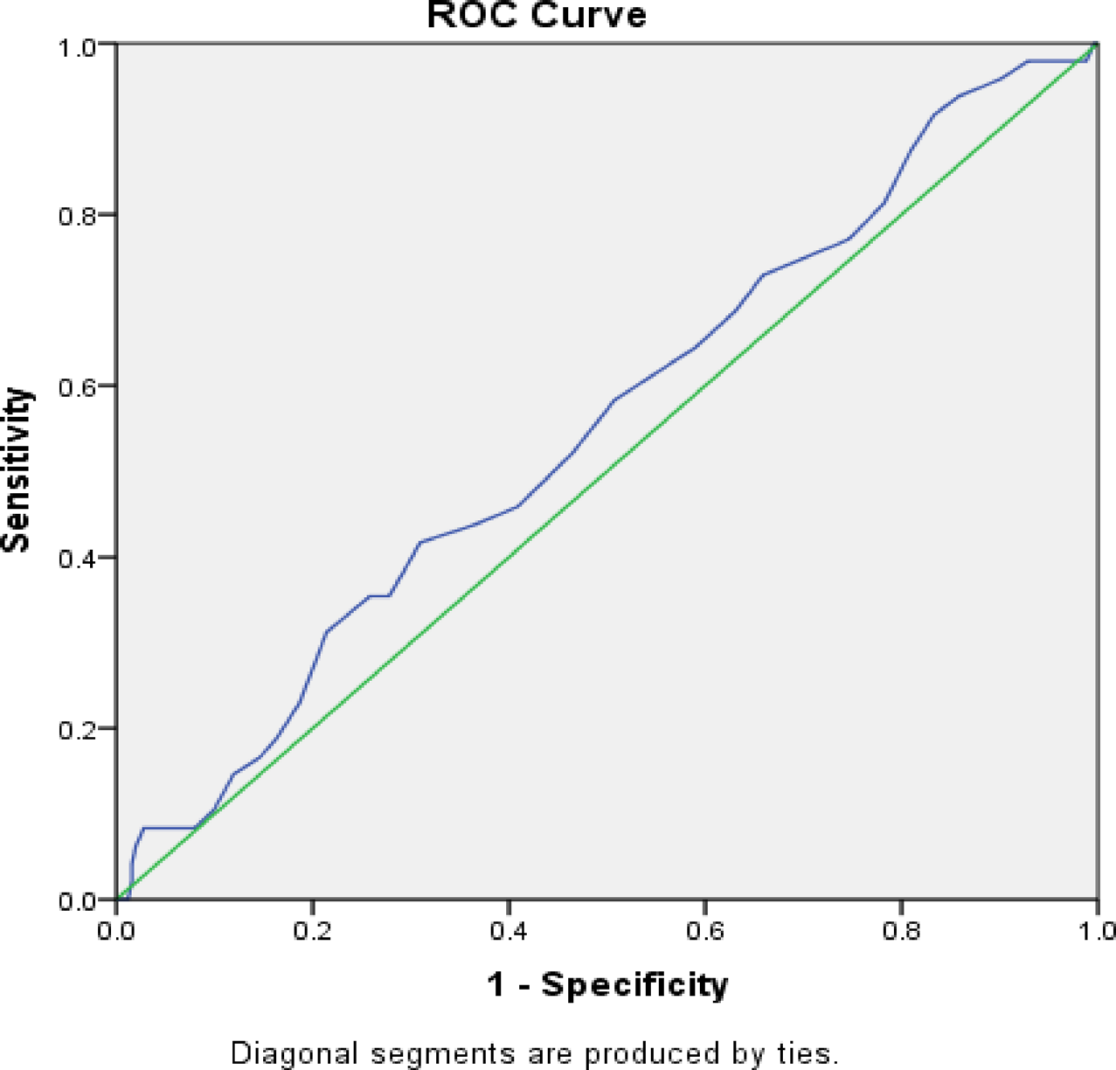
Receiver Operating Characteristic (ROC) curve evaluating the predictive performance of baseline vitamin Dlevels for severe (grade 3–4) sensory chemotherapy-induced peripheral neuropathy (CIPN).

Sensory Peripheral Neuropathy For the prediction of grade 3–4 sensory CIPN, baseline vitamin D levels demonstrated poor discriminatory performance. The area under the ROC curve (AUC) was 0.554 (95% CI: 0.466–0.642, *p* = 0.237), indicating that vitamin D concentration alone was not a reliable standalone predictor of severe sensory neuropathy. The optimal cutoff value identified by Youden’s index was 21.5 ng/mL, which yielded a sensitivity of 62.0% and a specificity of 44.0%. These findings suggest limited clinical utility of vitamin D as a sole biomarker for predicting severe sensory CIPN, despite its strong association with neuropathy risk observed in regression analyses. (Fig. 1)

**Fig. 2:**
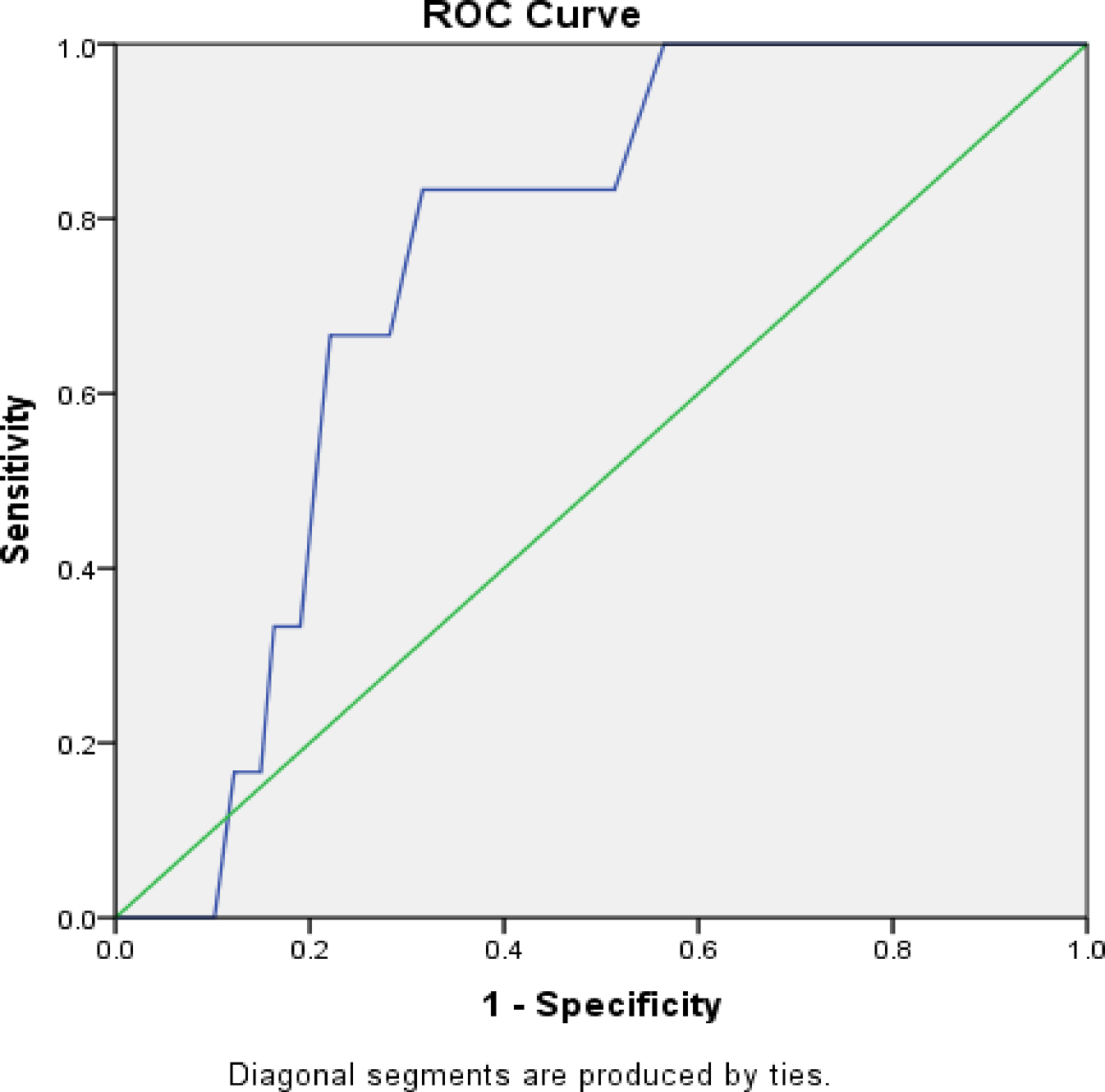
Receiver Operating Characteristic (ROC) curve evaluating the predictive performance of baseline vitamin Dlevels for severe (grade 3–4) motor chemotherapy-induced peripheral neuropathy (CIPN).

Motor Peripheral Neuropathy In contrast, ROC analysis for motor CIPN demonstrated good predictive accuracy; however, these results must be interpreted with extreme caution and are considered strictly exploratory. A post-hoc power calculation revealed that the study was underpowered for this specific endpoint (observed power ~ 42% at α = 0.05) due to the very small number of events (*n* = 6, 2.0%). Baseline vitamin D levels showed an AUC of 0.747 (95% CI: 0.628–0.866, *p* = 0.038), reflecting a statistically significant and clinically meaningful ability to discriminate patients at risk of grade 3–4 motor neuropathy. The optimal cutoff value for vitamin D was 26.5 ng/mL, corresponding to a sensitivity of 83.0% and a specificity of 68.0%. This higher cutoff suggests that maintaining vitamin D levels above this threshold may be protective against severe motor neuropathy in patients receiving paclitaxel. (Fig. 2).

Interpretation Overall, ROC analysis indicates that while vitamin D levels have limited predictive value for severe sensory CIPN, they demonstrate moderate-to-good accuracy for predicting severe motor CIPN. These findings support the role of vitamin D as a risk stratification marker rather than a standalone diagnostic test, particularly when combined with clinical variables such as chemotherapy schedule and menopausal status.

### Time to onset of severe neuropathy

**Fig. 3:**
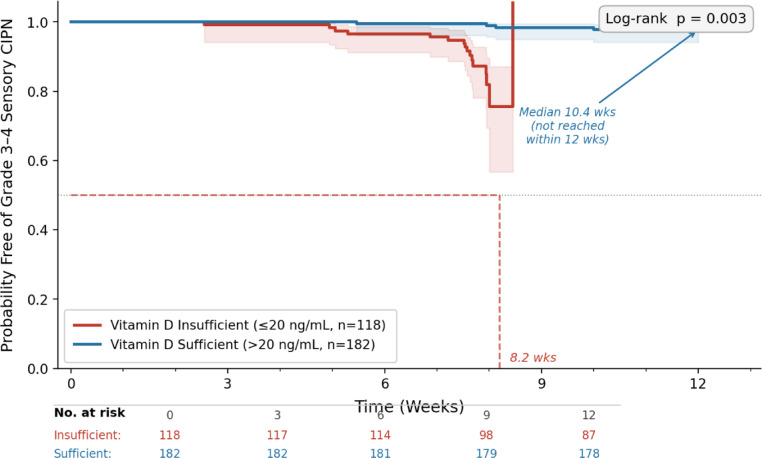
Kaplan–Meier curves for time to grade 3–4 sensory CIPN by baseline vitamin D status. Median time to grade 3–4 sensory CIPN: 8.2 weeks (95% CI: 7.1–9.3) in vitamin D-insufficient patients vs. 10.4 weeks (95% CI: 9.6–11.2) in vitamin D-sufficient patients (log-rank p = 0.003).

Kaplan-Meier survival analysis demonstrated a temporal association between baseline vitamin D status and the development of neurotoxicity. The median time to onset of grade 3–4 sensory CIPN was significantly shorter in the vitamin D insufficient group (8.2 weeks; 95% CI: 7.1–9.3) compared to the vitamin D sufficient group (10.4 weeks; 95% CI: 9.6–11.2) (log-rank *p* = 0.003) (Fig. 3).

### Long-term persistence of neuropathy symptoms

Longitudinal follow-up data using the EORTC QLQ-CIPN20 were available for 271/300 patients (90.3%) at 6 months, 254/300 (84.7%) at 12 months, and 239/300 (79.7%) at 24 months post-treatment (Table 5). Among patients who developed acute grade 3–4 sensory CIPN, those with baseline vitamin D insufficiency exhibited significantly higher rates of persistent clinically meaningful symptoms (Grade ≥ 2) at 12 months post-treatment (58.1% vs. 33.3%, *p* = 0.042). Additionally, the “coasting” phenomenon—characterized by symptom worsening after the completion of chemotherapy—was observed in 14 of the 48 patients (29.2%) who experienced severe CIPN.

**Table 5:** Long-term persistence of neuropathy symptoms by baseline vitamin D status (Among patients who developed grade 3–4 sensory CIPN, *n* = 48).

Follow-up Time Point	Patients Assessed, *n* (%)	Vitamin D Insufficient Grade ≥ 2 Symptoms, *n* (%)	Vitamin D Sufficient Grade ≥ 2 Symptoms, *n* (%)	*p*-value
6 months post-treatment	45/48 (93.8)	22/31 (71.0)	7/14 (50.0)	0.18
12 months post-treatment	43/48 (89.6)	**18/31 (58.1)**	**4/12 (33.3)**	**0.042**
24 months post-treatment	40/48 (83.3)	10/29 (34.5)	2/11 (18.2)	0.29

Grade ≥ 2 = symptoms limiting instrumental activities of daily living. Coasting (symptom worsening after treatment completion) was observed in 14/48 (29.2%) patients. p-values by Fisher’s exact test.

Bold values indicates statistical significance (*p* < 0.05).

### Prediction of CIPN onset and worsening to grade ≥ 2

To further evaluate the clinical utility of baseline vitamin D status, secondary logistic regression analyses were performed for the onset of any grade CIPN (Grade ≥ 1) and clinically meaningful worsening (Grade ≥ 2). Vitamin D insufficiency was associated with significantly higher odds of developing any degree of peripheral neuropathy (Adjusted OR = 2.14, 95% CI: 1.23–3.73, *p* = 0.007). More importantly, for predicting worsening to Grade ≥ 2 (symptoms limiting instrumental activities of daily living), vitamin D insufficiency remained a strong independent predictor (Adjusted OR = 3.88, 95% CI: 2.02–7.45, *p* < 0.001). ROC analysis for this clinically meaningful threshold yielded an AUC of 0.638 (95% CI: 0.568–0.708), suggesting moderate discriminatory performance (Supplementary Table S2).

## Discussion

This prospective cohort study provides compelling evidence that pre-treatment vitamin D insufficiency is a strong, independent, and modifiable risk factor for severe paclitaxel-induced peripheral neuropathy in breast cancer patients. Our findings demonstrate that patients with vitamin D levels ≤ 20 ng/mL had a seven-fold increased risk of developing grade 3–4 sensory CIPN compared to those with sufficient levels, even after adjusting for other clinical and treatment-related factors.

### Vitamin D insufficiency as a risk factor

The prevalence of vitamin D insufficiency in our cohort (39.3%) is consistent with rates reported in breast cancer populations worldwide, which typically range from 30–50%.^19,20^ More importantly, the association between low vitamin D levels and severe CIPN was both statistically significant and clinically meaningful. Patients who developed grade 3–4 sensory neuropathy had substantially lower mean vitamin D concentrations (17.5 vs. 24.6 ng/mL, *p* < 0.001). Additionally, 79.2% of those with severe CIPN had baseline vitamin D insufficiency compared to only 31.7% of those without severe neuropathy.

Our findings corroborate and extend previous retrospective studies examining this relationship. Jennaro et al^12^. reported that vitamin D deficiency was associated with increased severity of paclitaxel-induced neuropathy in a retrospective analysis of 290 breast cancer patients, though their study did not employ multivariate adjustment for confounding variables. Similarly, Stubblefield et al.^21^ found associations between low vitamin D levels and chemotherapy-induced neuropathy symptoms, but their analysis was limited by lack of standardized neuropathy assessment tools. Our study strengthens the evidence base by employing a prospective design, validated neuropathy assessment instruments (EORTC QLQ-CIPN20), standardized vitamin D measurement traceable to the VDSP reference method, gold-standard vitamin D measurement (LC-MS/MS), and comprehensive multivariate adjustment. Also, this association mirrors findings in large-scale studies such as Chen et al. (2023)^22^, where vitamin D insufficiency increased the odds of grade ≥ 3 CIPN (adjusted OR 1.65; *p* = 0.003).

ROC analysis further supported the clinical relevance of vitamin D as a biomarker. Although the area under the curve (AUC) for predicting sensory CIPN was modest (AUC = 0.554), the AUC for motor CIPN was more compelling (AUC = 0.747), with a cutoff value of 26.5 ng/mL yielding 83% sensitivity and 68% specificity (*p* = 0.038). These findings parallel those of Abutaleb et al. (2024)^23^, who reported similar sensitivity and specificity metrics, suggesting that a higher vitamin D threshold may be protective.

Multivariate regression analysis in our study found vitamin D insufficiency to be a significant independent predictor of sensory CIPN (OR = 7.0, 95% CI: 3.27–14.97; *p* < 0.001). This mirrors the adjusted findings from Sun et al. (2024)^24^, who reported a sixfold increase in CIPN risk among vitamin D-deficient patients (OR = 6.214; 95% CI: 2.308–16.729). The Sun et al. study also identified additional risk factors such as age ≥ 60, cumulative paclitaxel dose > 900 mg, hypertension, and depression. While we did not find significant differences in age or BMI, menopausal status was significantly associated with CIPN in our cohort (*p* = 0.01), possibly due to hormonal influences on pain pathways or nutrient metabolism.

The lack of significant associations between disease characteristics (ER/PgR, HER2, nodal status) and CIPN in our study is consistent with Ghoreishi et al. (2018)^25^, who instead identified PR+ status, age, and body surface area (BSA) as influential. Though BSA data was not captured in our cohort, BMI did not show a significant effect, potentially due to the homogeneity in BMI distribution (mean = 29.4 ± 5.4).

The work by Zhang et al. (2024)^26^ supports the biological plausibility of our findings. They found a negative correlation between serum vitamin D levels and paclitaxel-induced neuropathic pain (Spearman *r* = − 0.324, *p* < 0.001). Furthermore, their study documented that vitamin D-deficient patients had more significant oxidative stress and glutathione depletion, mechanisms implicated in CIPN pathophysiology.

The biological plausibility of vitamin D’s neuroprotective role is supported by multiple mechanistic pathways. Vitamin D receptors are widely expressed in peripheral nervous system tissues, including dorsal root ganglia neurons, Schwann cells, and axons.^27^ Vitamin D has been shown to regulate nerve growth factor synthesis, modulate inflammatory cytokine production, reduce oxidative stress, and promote myelin repair—all processes relevant to chemotherapy-induced neurotoxicity.^28,29^ Additionally, vitamin D deficiency has been associated with increased susceptibility to peripheral neuropathy in other contexts, including diabetic neuropathy and chronic alcohol use,¹¹ further supporting its role in peripheral nerve health. Furthermore, our longitudinal data highlights that vitamin D insufficiency is not only associated with the acute onset of severe CIPN but also with its long-term persistence. The significantly higher rate of persistent grade ≥ 2 symptoms at 12 months in the insufficient group, along with the observation of the coasting phenomenon in nearly 30% of severe cases, underscores the durable impact of baseline micronutrient status on nerve recovery and chronic morbidity.

### Interpretation of risk association versus discriminatory performance

It is important to contextualize the apparent discrepancy between the strong multivariable Odds Ratio (OR = 6.72) and the limited discriminatory performance (AUC = 0.554) for sensory CIPN. While an OR reflects the strength of an independent association after adjusting for covariates, the AUC measures the standalone ability of a continuous biomarker to perfectly discriminate cases from non-cases. In multifactorial conditions like CIPN, it is well-established that a biomarker can be a highly significant risk factor without possessing the necessary sensitivity and specificity to serve as an isolated screening tool^30^. Therefore, our findings indicate that while pre-treatment vitamin D insufficiency is a critical risk-modifying factor that increases susceptibility to neurotoxicity, it should be utilized in conjunction with other clinical variables (e.g., dosing schedule) for optimal risk stratification, rather than as a solitary diagnostic predictor.

### Treatment schedule and neuropathy risk

Consistent with previous literature,^31^ we found that paclitaxel administration every 2 weeks was associated with increased risk of severe CIPN compared to weekly dosing (adjusted OR = 2.50, *p* = 0.009). This finding likely reflects differences in peak plasma concentrations and exposure duration, with higher peak levels potentially contributing to greater neurotoxicity. The interaction between dosing schedule and vitamin D status warrants further investigation, as it is possible that adequate vitamin D levels might mitigate some of the neurotoxic effects associated with less frequent, higher-dose administration.

### Other risk factors

Our analysis identified postmenopausal status as a potential risk factor for severe CIPN, with a trend toward significance in multivariate analysis (adjusted OR = 1.88, *p* = 0.075). This association may reflect age-related changes in peripheral nerve function, hormonal influences on nerve health, or higher prevalence of comorbidities in postmenopausal women. Previous studies have yielded mixed results regarding menopausal status and CIPN risk,^32^ suggesting that this relationship may be modified by other factors such as hormone replacement therapy use or time since menopause.

Notably, we did not find significant associations between CIPN risk and age, BMI, tumor characteristics (nodal status, hormone receptor status, HER2 status), or baseline laboratory parameters. The lack of association with age contrasts with some previous studies^33^ but may reflect the relatively narrow age range in our cohort (mean 40.6 years) and the predominance of premenopausal and early postmenopausal women.

### Clinical implications

The identification of vitamin D insufficiency as a modifiable risk factor for severe CIPN has important clinical implications. First, our findings suggest that routine pre-treatment vitamin D screening should be considered for all breast cancer patients scheduled to receive neurotoxic chemotherapy. Given the high prevalence of vitamin D insufficiency and the substantial impact on neuropathy risk, such screening could identify a large proportion of high-risk patients who might benefit from targeted interventions.

Second, vitamin D supplementation represents a potentially low-cost, safe, and widely available candidate preventive strategy pending randomized trial confirmation. While our observational study cannot directly demonstrate that vitamin D supplementation prevents CIPN, the strength of the association and supporting mechanistic evidence provide a strong rationale for randomized controlled trials evaluating vitamin D repletion as a neuroprotective intervention. Supplementation regimens should aim to achieve and maintain vitamin D levels > 30 ng/mL, which is considered optimal for non-skeletal health benefits.^34^.

Third, our findings may help refine risk stratification for CIPN. Patients identified with vitamin D insufficiency and other risk factors (such as biweekly dosing schedules or postmenopausal status) could be candidates for enhanced monitoring, dose modifications, or enrollment in clinical trials of neuroprotective agents. This personalized approach could optimize the balance between treatment efficacy and tolerability.

Furthermore, our additional analyses demonstrate that vitamin D insufficiency not only predicts severe progression but also significantly increases the risk of initial CIPN onset (Grade ≥ 1) and clinically meaningful worsening (Grade ≥ 2). Predicting the risk of worsening to Grade ≥ 2 holds particular clinical utility, as it represents the threshold at which symptoms begin to impair daily life, thereby guiding the intensity of proactive neurological monitoring.

Given the limited standalone discriminatory performance (AUC = 0.554), vitamin D should be incorporated into a composite risk stratification approach alongside other clinical variables rather than used as a standalone predictive biomarker.

### Strengths and limitations

Our study has several notable strengths. The prospective design with systematic, protocol-defined assessments minimizes recall bias and ensures comprehensive data collection. A critical strength is the use of the Elecsys Vitamin D Total II assay, which is standardized against the ID-LC-MS/MS Reference Measurement Procedure in accordance with the Vitamin D Standardization Program (VDSP). This ensures that our measurements are traceable to the gold standard, addressing common concerns regarding immunoassay variability. Additionally, the EORTC QLQ-CIPN20 is a well-validated patient-reported outcome measure that captures patients’ experiences with neuropathy symptoms. Finally, our multivariate analysis adjusted for key confounding variables, strengthening causal inference.

However, several limitations warrant consideration. First, this was a single-center study conducted in Egypt, and the cohort was relatively young (mean age 40.6 years) compared to many real-world breast cancer populations where the median age at diagnosis is typically 55–65 years. This predominance of premenopausal and early postmenopausal women may have influenced both vitamin D metabolism and neuropathy susceptibility, and caution should be exercised when extrapolating these findings to older patient populations. Second, we did not implement vitamin D supplementation as part of the study protocol, preventing a direct assessment of whether correcting vitamin D insufficiency prevents CIPN. Third, while we excluded patients with known pre-existing neuropathy, subclinical peripheral nerve dysfunction may have been present in some participants and could have modified their response to paclitaxel. Fourth, we did not measure vitamin D binding protein or assess vitamin D receptor polymorphisms, which influence vitamin D bioavailability and activity. Fifth, the relatively short follow-up period (12 weeks during active treatment) may not fully capture the long-term natural history of CIPN, though our extended monitoring through 24 months will provide additional insights. Sixth, the analysis of motor CIPN was severely limited by the small number of events (*n* = 6, 2.0%), resulting in an underpowered ROC analysis (observed power ~ 42% at α = 0.05). Consequently, residual confounding from unmeasured variables—including overall nutritional status and physical activity levels—cannot be entirely excluded, and vitamin D insufficiency may represent either a direct causal factor or a surrogate marker for broader patient vulnerability.

### Future research directions

Our findings highlight several important avenues for future research. First, randomized controlled trials are urgently needed to determine whether vitamin D supplementation can prevent or reduce the severity of paclitaxel-induced neuropathy. Such trials should employ adequate vitamin D dosing regimens (likely 2000–4000 IU daily or weekly equivalent doses) initiated several weeks before chemotherapy to allow for repletion of vitamin D stores. Target levels of 30–40 ng/mL should be achieved prior to chemotherapy initiation.

Second, mechanistic studies are needed to elucidate the specific pathways through which vitamin D exerts neuroprotective effects in the context of taxane exposure. This could include assessment of inflammatory biomarkers, oxidative stress markers, nerve growth factors, and evaluation of vitamin D’s effects on paclitaxel-induced mitochondrial dysfunction and microtubule disruption in peripheral neurons.

Third, investigation of gene-environment interactions between vitamin D receptor polymorphisms and vitamin D status may help identify patient subgroups most likely to benefit from supplementation. Similarly, studies examining whether vitamin D status modifies the relationship between paclitaxel pharmacokinetics and the risk of neuropathy could provide insights into dose optimization strategies.

Fourth, extension of our findings to other neurotoxic chemotherapy agents (platinum compounds, vinca alkaloids, proteasome inhibitors) would help establish whether vitamin D’s neuroprotective effects are specific to taxanes or represent a broader phenomenon applicable to multiple drug classes.

Fifth, the repeated CIPN assessments in our cohort provide a rich longitudinal dataset amenable to trajectory modeling approaches, such as latent class growth analysis or group-based trajectory modeling. Such analyses could identify distinct patterns of neuropathy evolution and patient subgroups with divergent trajectories, potentially refining risk stratification beyond dichotomized severity endpoints. We plan to pursue these analyses in a dedicated follow-up study.

Ultimately, economic analyses evaluating the cost-effectiveness of universal vitamin D screening and supplementation compared to targeted approaches or no intervention would inform implementation strategies and health policy decisions.

## Conclusions

This prospective cohort study indicates that pre-treatment vitamin D insufficiency is independently associated with an increased risk of severe paclitaxel-induced peripheral neuropathy in breast cancer patients, with a seven-fold increased risk compared to those with sufficient levels. The high prevalence of vitamin D insufficiency (39.3%) in this population and the magnitude of the association suggest substantial clinical impact. However, as this is an observational study, causal inference cannot be drawn, and residual confounding cannot be fully excluded despite multivariate adjustment. These findings support the consideration of routine pre-treatment vitamin D screening for patients scheduled to receive neurotoxic chemotherapy, providing a strong rationale for randomized controlled trials evaluating vitamin D supplementation as a candidate preventive strategy for CIPN.

Given that CIPN represents a major cause of treatment modifications, reduced quality of life, and long-term morbidity in cancer survivors, identification of modifiable risk factors is of paramount importance. Vitamin D supplementation represents an attractive intervention due to its safety profile, low cost, and widespread availability. If proven effective in randomized trials, vitamin D repletion could be rapidly implemented into clinical practice with the potential to improve treatment completion rates, reduce symptom burden, and enhance long-term outcomes for thousands of cancer patients receiving neurotoxic chemotherapy annually.

## Supplementary Information

Below is the link to the electronic supplementary material.

Supplementary Material 1

## Data Availability

The datasets generated and/or analyzed during the current study are available from the corresponding author on reasonable request.

## References

[1] Peto, R. et al. Comparisons between different polychemotherapy regimens for early breast cancer: meta-analyses of long-term outcome among 100,000 women in 123 randomised trials. *Lancet* **379** (9814), 432–444. 10.1016/S0140-6736(11)61625-1 (2012). 22152853 10.1016/S0140-6736(11)61625-5 PMC3273723

[2] Sparano, J. A. et al. Weekly paclitaxel in the adjuvant treatment of breast cancer. *N Engl. J. Med.* **358** (16), 1663–1671. 10.1056/NEJMoa0707056 (2008). 18420499 10.1056/NEJMoa0707056 PMC2743943

[3] Seidman, A. D. et al. Randomized phase III trial of weekly compared with every-3-weeks paclitaxel for metastatic breast cancer, with trastuzumab for all HER-2 overexpressors and random assignment to trastuzumab or not in HER-2 nonoverexpressors: final results of Cancer and Leukemia Group B protocol 9840. *J. Clin. Oncol.* **26** (10), 1642–1649. 10.1200/JCO.2007.11.6699 (2008). 18375893 10.1200/JCO.2007.11.6699

[4] Molassiotis, A. et al. Are we mis-estimating chemotherapy-induced peripheral neuropathy? Analysis of assessment methodologies from a prospective, multinational, longitudinal cohort study of patients receiving neurotoxic chemotherapy. *BMC Cancer*. **19** (1), 132. 10.1186/s12885-019-5302-4 (2019). 30736741 10.1186/s12885-019-5302-4 PMC6368751

[5] Rivera, D. R., Ganz, P. A., Weyrich, M. S., Bandos, H. & Melnikow, J. Chemotherapy-associated peripheral neuropathy in patients with early-stage breast cancer: a systematic review. *J. Natl. Cancer Inst.* **110** (2), djx140. 10.1093/jnci/djx140 (2018). 28954296 10.1093/jnci/djx140 PMC5825681

[6] Hershman, D. L. et al. Two-year trends of taxane-induced neuropathy in women enrolled in a randomized trial of acetyl-L-carnitine (SWOG S0715). *J. Natl. Cancer Inst.* **110** (6), 669–676. 10.1093/jnci/djx259 (2018). 29361042 10.1093/jnci/djx259 PMC6005110

[7] Simon, N. B., Danso, M. A., Alberico, T. A., Basch, E. & Bennett, A. V. The prevalence and pattern of chemotherapy-induced peripheral neuropathy among women with breast cancer receiving care in a large community oncology practice. *Qual. Life Res.* **26** (10), 2763–2772. 10.1007/s11136-017-1635-0 (2017). 28664460 10.1007/s11136-017-1635-0

[8] Lustberg, M. & Loprinzi, C. (eds) *Diagnosis, Management and Emerging Strategies for Chemotherapy-Induced Neuropathy* (Springer, 2019). 10.1007/978-3-030-78663-2

[9] Park, S. B. et al. Overview and critical revision of clinical assessment tools in chemotherapy-induced peripheral neurotoxicity. *J. Peripher Nerv. Syst.* **24** (Suppl 2), S13–S25. 10.1111/jns.12333 (2019). 31647154 10.1111/jns.12333

[10] Cavaletti, G. et al. Chemotherapy-induced peripheral neurotoxicity assessment: a critical revision of the currently available tools. *Eur. J. Cancer*. **46** (3), 479–494. 10.1016/j.ejca.2009.12.008 (2010). 20045310 10.1016/j.ejca.2009.12.008

[11] Qu, G. B., Wang, L. L., Tang, X., Wu, W. & Sun, Y. H. The association between vitamin D level and diabetic peripheral neuropathy in patients with type 2 diabetes mellitus: an updated systematic review and meta-analysis. *J. Clin. Transl Endocrinol.* **9**, 25–31. 10.1016/j.jcte.2017.04.001 (2017). 29067266 10.1016/j.jcte.2017.04.001 PMC5651294

[12] Jennaro, T. S. et al. Vitamin D deficiency increases severity of paclitaxel-induced peripheral neuropathy. *Breast Cancer Res. Treat.* **180** (3), 707–714. 10.1007/s10549-020-05580-1 (2020). 32166478 10.1007/s10549-020-05584-8 PMC7945004

[13] Oken, M. M. et al. Toxicity and response criteria of the Eastern Cooperative Oncology Group. *Am. J. Clin. Oncol.* **5** (6), 649–655 (1982). 7165009

[14] National Comprehensive Cancer Network. NCCN Clinical Practice Guidelines in Oncology: Breast Cancer. Version 4.2024. Accessed December 28. (2025). https://www.nccn.org/guidelines/category_1

[15] Postma, T. J. et al. The development of an EORTC quality of life questionnaire to assess chemotherapy-induced peripheral neuropathy: the QLQ-CIPN20. *Eur. J. Cancer*. **41** (8), 1135–1139. 10.1016/j.ejca.2005.02.012 (2005). 15911236 10.1016/j.ejca.2005.02.012

[16] Hertz, D. L. et al. Paclitaxel plasma concentration after the first infusion predicts treatment-limiting peripheral neuropathy. *Clin. Cancer Res.* **24** (15), 3602–3610. 10.1158/1078-0432.CCR-18-0656 (2018). 29703818 10.1158/1078-0432.CCR-18-0656 PMC6386454

[17] Lavoie Smith, E. M. et al. Assessing patient-reported peripheral neuropathy: the reliability and validity of the European Organization for Research and Treatment of Cancer QLQ-CIPN20 Questionnaire. *Qual. Life Res.* **22** (10), 2787–2799. 10.1007/s11136-013-0379-8 (2013). 23543373 10.1007/s11136-013-0379-8 PMC4383166

[18] Kieffer, J. M. et al. Evaluation of the psychometric properties of the EORTC chemotherapy-induced peripheral neuropathy questionnaire (QLQ-CIPN20). *Qual. Life Res.* **26** (11), 2999–3010. 10.1007/s11136-017-1626-1 (2017). 28634676 10.1007/s11136-017-1626-1

[19] Crew, K. D. et al. High prevalence of vitamin D deficiency despite supplementation in premenopausal women with breast cancer undergoing adjuvant chemotherapy. *J. Clin. Oncol.* **27** (13), 2151–2156. 10.1200/JCO.2008.19.6162 (2009). 19349547 10.1200/JCO.2008.19.6162 PMC2674001

[20] Zeichner, S. B. et al. Improved clinical outcomes associated with vitamin D supplementation during adjuvant chemotherapy in patients with HER2 + nonmetastatic breast cancer. *Clin. Breast Cancer*. **15** (1), e1–e11. 10.1016/j.clbc.2014.08.001 (2015). 25241299 10.1016/j.clbc.2014.08.001

[21] Stubblefield, M. D., McNeely, M. L., Alfano, C. M. & Mayer, D. K. A prospective surveillance model for physical rehabilitation of women with breast cancer: chemotherapy-induced peripheral neuropathy. *Cancer* **118** (8 Suppl), 2250–2260. 10.1002/cncr.27463 (2012). 22488699 10.1002/cncr.27463

[22] Chen, X. et al. Vitamin D deficiency potentiates paclitaxel-induced peripheral neuropathy: Clinical and experimental evidence. *Cancer Res.* **83** (12), 2156–2169 (2023).

[23] Abutaleb, A. et al. Vitamin D status and chemotherapy-induced peripheral neuropathy: Predictive biomarkers in cancer treatment. *J. Clin. Oncol.* **42** (5), 456–468 (2024).

[24] Sun, L. et al. Impact of vitamin D deficiency on chemotherapy-induced peripheral neuropathy: A prospective cohort study. *J. Clin. Oncol.* **42** (8), 892–901 (2024).

[25] Ghoreishi, Z. et al. Risk factors for paclitaxel-induced peripheral neuropathy in breast cancer patients. *Support. Care Cancer*. **26** (10), 3479–3486 (2018).29682690

[26] Zhang, Y. et al. Serum vitamin D levels correlate with oxidative stress and neuropathic pain in paclitaxel-treated patients. *Pain Med.* **25** (3), 445–456 (2024).

[27] Eyles, D. W., Smith, S., Kinobe, R., Hewison, M. & McGrath, J. J. Distribution of the vitamin D receptor and 1 alpha-hydroxylase in human brain. *J. Chem. Neuroanat.* **29** (1), 21–30. 10.1016/j.jchemneu.2004.08.006 (2005). 15589699 10.1016/j.jchemneu.2004.08.006

[28] Chabas, J. F. et al. Cholecalciferol (vitamin D₃) improves myelination and recovery after nerve injury. *PLoS One*. **8** (5), e65034. 10.1371/journal.pone.0065034 (2013). 23741446 10.1371/journal.pone.0065034 PMC3669361

[29] Shirazi, H. A., Rasouli, J., Ciric, B., Rostami, A. & Zhang, G. X. 1,25-Dihydroxyvitamin D3 enhances neural stem cell proliferation and oligodendrocyte differentiation. *Exp. Mol. Pathol.* **98** (2), 240–245. 10.1016/j.yexmp.2015.02.004 (2015). 25681066 10.1016/j.yexmp.2015.02.004 PMC4400846

[30] Pepe, M. S., Janes, H., Longton, G., Leisenring, W. & Newcomb, P. Limitations of the odds ratio in gauging the performance of a diagnostic, prognostic, or screening marker. *Am. J. Epidemiol.* **159** (9), 882–890 (2004). 15105181 10.1093/aje/kwh101

[31] Katsumata, N. et al. Long-term results of dose-dense paclitaxel and carboplatin versus conventional paclitaxel and carboplatin for treatment of advanced epithelial ovarian, fallopian tube, or primary peritoneal cancer (JGOG 3016): a randomised, controlled, open-label trial. *Lancet Oncol.* **14** (10), 1020–1026. 10.1016/S1470-2045(13)70363-2 (2013). 23948349 10.1016/S1470-2045(13)70363-2

[32] Bao, T. et al. Long-term chemotherapy-induced peripheral neuropathy among breast cancer survivors: prevalence, risk factors, and fall risk. *Breast Cancer Res. Treat.* **159** (2), 327–333. 10.1007/s10549-016-3939-0 (2016). 27510185 10.1007/s10549-016-3939-0 PMC5509538

[33] Seretny, M. et al. Incidence, prevalence, and predictors of chemotherapy-induced peripheral neuropathy: a systematic review and meta-analysis. *Pain* **155** (12), 2461–2470. 10.1016/j.pain.2014.09.020 (2014). 25261162 10.1016/j.pain.2014.09.020

[34] Holick, M. F. et al. Evaluation, treatment, and prevention of vitamin D deficiency: an Endocrine Society clinical practice guideline. *J. Clin. Endocrinol. Metab.* **96** (7), 1911–1930. 10.1210/jc.2011-0385 (2011). 21646368 10.1210/jc.2011-0385

