# Supplementary material for: Pre-treatment vitamin D insufficiency predicts severe paclitaxel-induced sensory neuropathy in breast cancer patients: a prospective cohort study
Source: Sci Rep. 2026 May 5;16:14282. doi: 10.1038/s41598-026-50367-8 (PMC13144410; doi:10.1038/s41598-026-50367-8)
Supplement: Supplementary file 1 — Supplementary Material 1 [file 41598_2026_50367_MOESM1_ESM.docx]

**Supplementary Material**

**Journal:** Scientific Reports

**Title:** Pre-treatment Vitamin D Insufficiency Predicts Severe Paclitaxel-Induced Sensory Neuropathy in Breast Cancer Patients: A Prospective Cohort Study

**Authors:** Amany M Elfeky, Muhammad I. El-Masry, Amr A Mahmoud, Sara A. Amin, Rana El Falah, and Mo’men M. Saadoun

**Corresponding Author:**

Mo’men Mahmoud Saadoun

Lecturer of Clinical Pathology, Faculty of Medicine, Kafrelsheikh University, Kafrelsheikh, Egypt

Email: momen.saadoun@med.kfs.edu.eg

**Table of Contents for Supplementary Material:**

- **Supplementary Table S1:** Sensitivity Analysis: Multivariate Logistic Regression for Grade 3–4 Sensory CIPN Restricted to the Weekly Paclitaxel Cohort (n = 153)
- **Supplementary Table S2:** Logistic Regression Analyses for Prediction of Any CIPN Onset (Grade ≥1) and Clinically Meaningful Worsening to Grade ≥2 by Baseline Vitamin D Status

**Supplementary Table S1. Sensitivity Analysis: Multivariate Logistic Regression for Grade 3–4 Sensory CIPN Restricted to the Weekly Paclitaxel Cohort (n = 153)**

| **Variable** | **Adjusted OR (95% CI)** | **p-value** |
| --- | --- | --- |
| **Vitamin D insufficiency (≤20 ng/mL)** | 5.84 (2.11–11.45) | **0.004** |
| **Postmenopausal status** | 1.62 (0.78–3.21) | 0.180 |
| **Cumulative delivered dose (mg/m²)** | 1.002 (0.990–1.005) | 0.450 |
| **Delivered dose intensity (%)** | 0.96 (0.85–1.08) | 0.320 |

**Supplementary Table S2. Logistic Regression Analyses for Prediction of Any CIPN Onset (Grade ≥1) and Clinically Meaningful Worsening to Grade ≥2 by Baseline Vitamin D Status**

***Panel A. Prediction of Any CIPN Onset (Grade ≥1) — Multivariate Logistic Regression (N = 300)***

| **Variable** | **Grade ≥1 CIPN (n=240)** | **No CIPN (n=60)** | **Adjusted OR (95% CI)** | **p-value** |
| --- | --- | --- | --- | --- |
| **Vitamin D insufficiency (≤20 ng/mL), n (%)** | **100 (83.3)** | **18 (30.0)** | **2.14 (1.23–3.73)** | **0.007** |
| **Paclitaxel every 2 weeks, n (%)** | **132 (55.0)** | 15 (25.0) | **2.08 (1.08–4.01)** | **0.029** |
| Postmenopausal status, n (%) | 136 (56.7) | 27 (45.0) | 1.54 (0.83–2.86) | 0.17 |
| Age (per year increase) | — | — | 1.02 (0.98–1.05) | 0.31 |
| BMI (per kg/m² increase) | — | — | 1.00 (0.94–1.06) | 0.99 |

*OR = odds ratio; CI = confidence interval. Bold = statistically significant (p < 0.05). Grade ≥1 CIPN = any neuropathy onset (n=240 events). Model adjusted for age, BMI, menopausal status, treatment schedule, cumulative delivered dose, and delivered dose intensity.*

***Panel B. Prediction of Clinically Meaningful Worsening to Grade ≥2 CIPN — Multivariate Logistic Regression (N = 300)***

ROC analysis for vitamin D as predictor of grade ≥2 CIPN: AUC = 0.638 (95% CI: 0.568–0.708, p < 0.001); optimal cutoff 20.5 ng/mL; sensitivity 71.2%, specificity 58.4%.

| **Variable** | **Grade ≥2 CIPN (n=237)** | **Grade <2 CIPN (n=63)** | **Adjusted OR (95% CI)** | **p-value** |
| --- | --- | --- | --- | --- |
| **Vitamin D insufficiency (≤20 ng/mL), n (%)** | **105 (88.2)** | **13 (20.6)** | **3.88 (2.02–7.45)** | **<0.001** |
| **Paclitaxel every 2 weeks, n (%)** | **138 (58.2)** | 9 (14.3) | **2.31 (1.04–5.13)** | **0.039** |
| **Cumulative delivered dose (mg/m²)** | Mean 834.1 | Mean 784.2 | **1.003 (1.001–1.006) per mg/m²** | **0.018** |
| Postmenopausal status, n (%) | 141 (59.5) | 22 (34.9) | 1.62 (0.88–2.99) | 0.12 |
| Age (per year increase) | — | — | 1.02 (0.99–1.06) | 0.19 |
| BMI (per kg/m² increase) | — | — | 1.01 (0.95–1.07) | 0.81 |

*OR = odds ratio; CI = confidence interval. Bold = statistically significant (p < 0.05). Grade ≥2 = symptoms limiting instrumental activities of daily living. Model adjusted for age, BMI, menopausal status, treatment schedule, cumulative delivered dose, and delivered dose intensity. ROC AUC for vitamin D predicting grade ≥2 CIPN = 0.638 (95% CI: 0.568–0.708), suggesting moderate discriminatory performance with potential clinical utility for guiding monitoring intensity.*
